# Can we avoid casting for suspected scaphoid fractures? A multicenter randomized controlled trial

**DOI:** 10.1186/s10195-025-00822-5

**Published:** 2025-03-05

**Authors:** Abigael Cohen, Max Reijman, Gerald A. Kraan, Sara J. Baart, Jan A. N. Verhaar, Joost W. Colaris, the SUSPECT study group, Peer van der Zwaal, Merel van Loon, Steven J Rhemrev, Daphne A van Rijssel, Mark R de Vries, Flip van Beek, Sander Mol, Tjebbe Hagenaars, Els H Jansen, Alexander P A Greeven, Lenneke C M Scholtens, Ruud L M Deijkers, Niels W L Schep, Akkie N Ringburg, Milko M M Bruijninckx, Kirsten F van Meerten, Willem-Maarten P F Bosman

**Affiliations:** 1 Department of Orthopaedics and Sport Medicine, Erasmus MC University Medical Center, PO box 2040, 3000 CA Rotterdam, The Netherlands; 2 Department of Orthopaedic Surgery, Reinier Haga Orthopaedic Center, Zoetermeer, The Netherlands; 3 Department of Biostatistics, Erasmus MC University Medical Center, Rotterdam, The Netherlands

**Keywords:** Adults, Fractures, Bone, Scaphoid bone, Wrist injuries, Patient-reported outcome measures

## Abstract

**Background:**

In suspected scaphoid fractures with normal initial radiographs, the usual care is casting, but only 10% of patients have scaphoid fractures. To reduce overtreatment, we evaluated whether bandaging, instead of casting, resulted in noninferior functional outcomes.

**Patients and methods:**

We included adults with suspected scaphoid fractures and normal initial radiographs at the emergency department in our multicenter randomized controlled trial. Patients were randomized to 3-day bandaging or 2-week casting. Questionnaires, physical examination, and radiographs were performed at 2 weeks and 1 year. Additional questionnaires were sent after inclusion, 6 weeks, and 3 months. Our primary outcome was the adjusted estimated difference between groups of the Quick Disabilities of the Arm, Shoulder, and Hand (QDASH) score at 3 months (natural logarithm of the margin of noninferiority = 2.0). Secondary outcomes included the QDASH score, Patient-Rated Hand/Wrist Evaluation Score, visual analog scale pain, wrist range of motion, patient satisfaction, and complications during follow-up.

**Results:**

Of the 180 patients (91 bandaging and 89 casting), 16 had scaphoid fractures and there were no scaphoid nonunions. Functional outcome in the bandaging group was noninferior at 3 months compared with the casting group [adjusted estimated difference QDASH score 0.30 (95% CI 0.02–0.62)]. All other patient-reported function and pain scores were not significantly different between groups. Range of motion at 2 weeks was better in the bandaging group, and they were more satisfied with the treatment than the casting group.

**Conclusions:**

Casting for suspected scaphoid fractures but normal initial radiographs can be avoided because bandaging seems to be an alternative treatment option when patients are reevaluated after 2 weeks.

*Level of evidence* Level II.

*Trial registration* Trial registered at the Trialregister on 2018-02-28 on www.trialregister.nl,

NTR7164

**Supplementary Information:**

The online version contains supplementary material available at 10.1186/s10195-025-00822-5.

## Introduction

Although only 10% of patients with suspected scaphoid fractures (but normal initial radiographs) actually have an occult scaphoid fracture on follow-up, the usual care is treatment with 2 weeks casting before reexamination [1]. This means that 90% of patients are overtreated [2, ]. Patients who present at the emergency department after a trauma, with pain over the scaphoid and normal initial radiographs, are suspected to have scaphoid fractures. Occult scaphoid fracture are scaphoid fractures that become visible during follow-up imaging modalities in these patients.

Scaphoid fractures are the most common carpal fractures, occurring mainly in young, working adults [3]. Patients present to the family doctor or emergency department with traumatic radial-sided wrist pain. Identifying scaphoid fractures is challenging because initial radiographs can be normal and occult fractures become apparent at follow-up [4].

The rationale behind casting all patients with suspected scaphoid fractures is that untreated fractures can fail to unite. Scaphoid nonunions require surgery and can lead to wrist osteoarthritis [5]. When immobilization is started within 4 weeks of trauma there is no increase in nonunion rates [6].

To reduce unnecessary casting, patients with suspected scaphoid fractures could be treated with bandaging instead of casting. A single trial from 1988 has examined this and found no differences in healing complications or pain between the two groups [7]. Unfortunately, this study failed to report any information regarding randomization, measurement of the outcomes, a specified analysis plan, or duration of follow-up. These serious methodological shortcomings, and potential for bias, mean that these results are questionable. Therefore, we conducted a new high quality randomized controlled trial with 1-year follow-up to evaluate functional outcomes between patients with a suspected scaphoid fracture (and normal initial radiographs) treated with bandaging compared with casting. We hypothesized that bandaging results in noninferior functional outcome at 3 months compared with casting.

## Patients and methods

### Study design and setting

We performed the SUSPECT study (clinically SUSPEcted SCaphoid fracTure: treatment with supportive bandage or cast study), which was a pragmatic, parallel-group, multicenter, open-label, noninferiority randomized controlled trial conducted in the Netherlands with 1-year follow-up. The medical ethics committee of the Erasmus MC University Medical Center approved the study protocol prior to study commencement and all subsequent amendments. Local institutional review boards granted approval before study onset. Trial registered at 2018-02-28 on www.trialregister.nl, NTR7164. Written informed consent from all patients was obtained prior to inclusion. The study was performed in accordance with the Declaration of Helsinki and Good Clinical Practice Guidelines. The design of the SUSPECT study was published previously [8]. Owing to Dutch privacy law, we were not allowed to screen the electronic patient reports to check the number of eligible patients not participating in our study.

### Selection of participants

Patients were recruited at the emergency departments (ED) of nine hospitals from June 2018 to January 2020. The physicians provided study information if patients were eligible to participate. The inclusion and exclusion criteria are provided in Table 1. Patients fulfilling the inclusion criteria, and not the exclusion criteria, are patients with suspected scaphoid fractures in this study.

**Table 1:** Inclusion and exclusion criteria

Inclusion criteria	Exclusion criteria
• ≥ 18 years • Trauma within 48 h • Anatomical snuff box or scaphoid tubercle tenderness • Normal wrist radiographs with minimally three scaphoid specific views (reported by physician and radiologist)	• Concomitant injury of the ipsilateral extremity that needed movement restriction by cast or bandage • Inability to complete study forms owing to insufficient command of the Dutch language • If the supervising radiologist reported retrospectively a scaphoid fracture on the initial radiographs

### Randomization and blinding

Patients who were included were immediately electronically randomized by their physician at the ED to bandaging or casting using Castor electronic data capture (Castor EDC) [9]. Variable block sizes (2, 4, or 6) in a 1:1 ratio were used to conceal random treatment allocation. Randomization was stratified per hospital. Treatment was started directly after randomization.

The physician, patient, and researcher were not blinded, but the assessment of the radiographs and the statistical analysis was performed blinded to the treatment allocation.

### Interventions

**Fig. 1:**
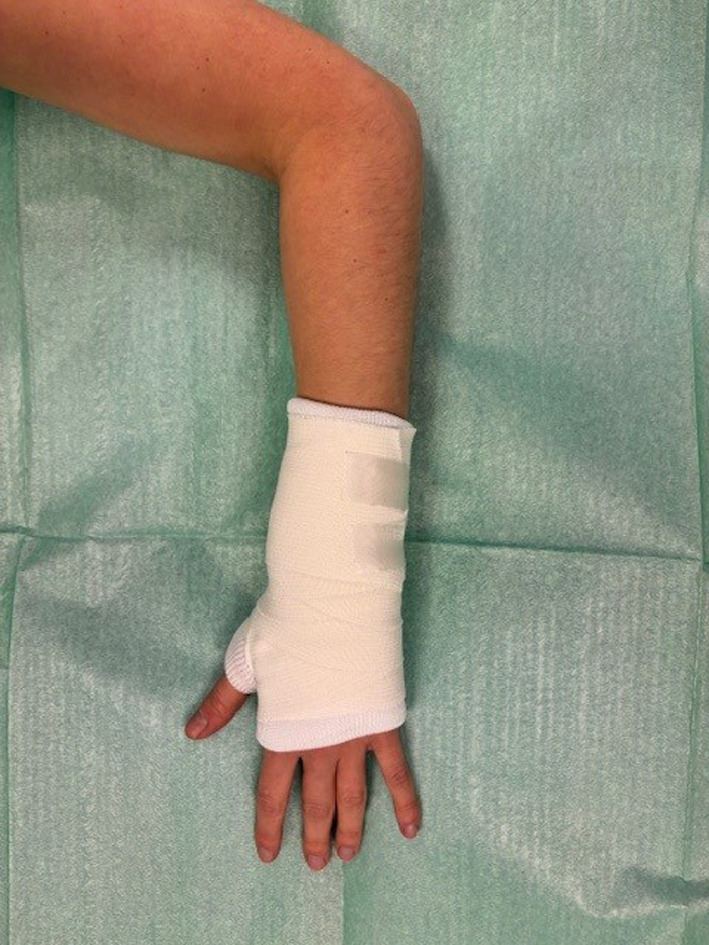
Below-elbow supportive bandage

Patients in the bandaging group received below-elbow supportive bandaging around the wrist, without the thumb (Fig. 1). After 3 days, patients were allowed to remove the bandage and move the wrist, guided by the amount of pain. Patients assigned to the casting group, received below-elbow casting according to the local hospital protocol (circular or splint, with or without thumb) until the outpatient department appointment after 2 weeks. Crossover was permitted in both groups.

In keeping with usual care in the Netherlands, a physician re-examined all patients after 2 weeks, and radiographs with at least three scaphoid specific views were obtained. The physician determined the diagnosis, treatment, and follow-up without researcher interference. Patients diagnosed with scaphoid fractures were treated following hospital protocols.

For study purposes, the researchers performed a physical examination after 2 weeks and 1 year, with wrist radiographs after 1 year, to evaluate fracture union. Questionnaires were emailed by Castor EDC after inclusion, and at 2 weeks, 6 weeks, 3 months, and 1 year, and when no email address was available, paper forms were posted to patients. Patients who did not complete questionnaires were contacted by telephone.

### Outcomes

The primary outcome was functional outcome measured with the Quick Disabilities of the Arm, Shoulder and Hand (QDASH) [10] questionnaire at 3 months after inclusion. The QDASH score is based on 11 questions about physical function and the pain of both arms during the last week (score 0–100; optimal score 0).

Secondary outcomes concerning functional outcomes and pain included (1) the QDASH score at the other follow-up points (inclusion, 2 weeks, 6 weeks and 1 year); (2) the patient-rated wrist/hand evaluation (PRWHE) [11] score (score 0–100; optimal score 0) at all follow-up points; (3) the amount of pain scored on the visual analog scale (VAS) at rest and during movement at all follow-up points (score 0–10; optimal score 0); (4) physical examination after 2 weeks and 1 year of the uninjured wrist compared with the injured wrist (lower score indicates better physical functioning). This consisted of measuring the range of motion of the wrist (palmar and dorsal flexion, ulnar and radial deviation, supination, and pronation), grip strength, finger motion compromising the finger-to-palm distance [12], and the Kapandji score (thumb opposition) [13]; and (5) occult scaphoid fractures identified during follow-up according to the treating physician on the basis of their interpretation of the physical examination, radiographs, and/or further imaging.

Patient satisfaction after 2 weeks and 3 months was assessed using two questions: “How satisfied are you with the treatment you received at the emergency department?” (score 0–10; optimal score 10) and “Would you have preferred the other treatment at the ED? (yes or no).”

The proportion of (serious) adverse events as treatment-related problems, complex regional pain syndrome diagnosed by a physician, persisting pain after 6 weeks, 3 months, or 1 year [Question: “Do you still have a painful wrist” (yes or no question)]. surgically treated scaphoid fracture, compartment syndrome, and scaphoid nonunion were evaluated during follow-up by questionnaires, medical records, and wrist radiographs after 1 year.

### Analysis

To demonstrate with 90% power [a one-sided alpha of 0.025 and standard deviation (SD) of 14] that bandaging compared with casting results in a noninferior QDASH score at 3 months, we needed 148 patients [14, 15]. When accounting for 15% lost to follow-up, 180 patients were required.

All outcomes were primarily analyzed as-randomized (intention-to-treat). Normally distributed continuous outcomes were analyzed with linear mixed effects models. The model assumptions were violated when analyzing the QDASH, PRWHE and VAS pain outcomes, because many patients reported the best possible outcome (zero) resulting in an overall nonnormal distribution of data. These outcomes were analyzed with a hurdle mixed effects model, that can handle data with excess zeroes, as a two-part mixed model with a zero and nonzero part (which was changed before data was unblinded) [16, 17]. For the nonzero part, a log-normal distribution of the data was assumed. The same fixed effects and random intercepts were added to both the zero and nonzero parts of the model.

The fixed effects for all mixed models were; randomized treatment, time, the interaction term of time by treatment, age, sex, and the presence of comorbidities that influence the function of the arms. In the linear mixed model, a three-level structure of patients within hospitals was modeled through the random effects, using an unstructured variance–covariance matrix. In the hurdle models, only random intercepts on patient-level were included. More complex hierarchical structures were not feasible in the estimation of the models.

For all outcomes, one model with all available data until 3 months was created to predict outcomes up to 3 months. To predict the outcomes at 1 year, a second model was constructed using data from all time points. For physical examination, we created one model including the 2-week and 1-year measurements. Adjusted estimated means and adjusted estimated differences with their 95% confidence intervals (95% CI) were predicted by the coefficients of the linear model, or by calculating marginal coefficients of the two parts of the hurdle model. The marginal coefficients in the hurdle model correspond to the average mixture outcome, meaning a combination of the zero and nonzero parts of the outcome.

For our primary outcome (noninferiority), QDASH at 3 months, the margin of noninferiority (7.5 points) was calculated as 50% of the margin of clinical minimal important difference of QDASH [14]. Since a hurdle model assumes a log-normal distribution of the data, the natural logarithm of the margin of noninferiority (2.0) is used in this study. Therefore, the 95% CI of the adjusted estimated difference between the two groups should not include 2.0.

For the other outcomes we performed two-sided testing, as we did not state a margin of noninferiority prior to the start of the study. A *p*-value < 0.05 was considered significant or (when a hurdle model was used) if the 95% CI of the adjusted estimated differences between the two groups did not include 0 on the log-normal scale. Additional as-treated analyses (per-protocol) were performed with similar models as the as-randomized analysis.

## Results

### Characteristics of study subjects

Of the 185 patients randomized between June 2018 and January 2020, 5 were excluded because they withdrew before data collection started. All other patients were included in our primary analysis, including six patients (3%) who did not attend hospital follow-up but responded to at least one questionnaire.

**Fig. 2:**
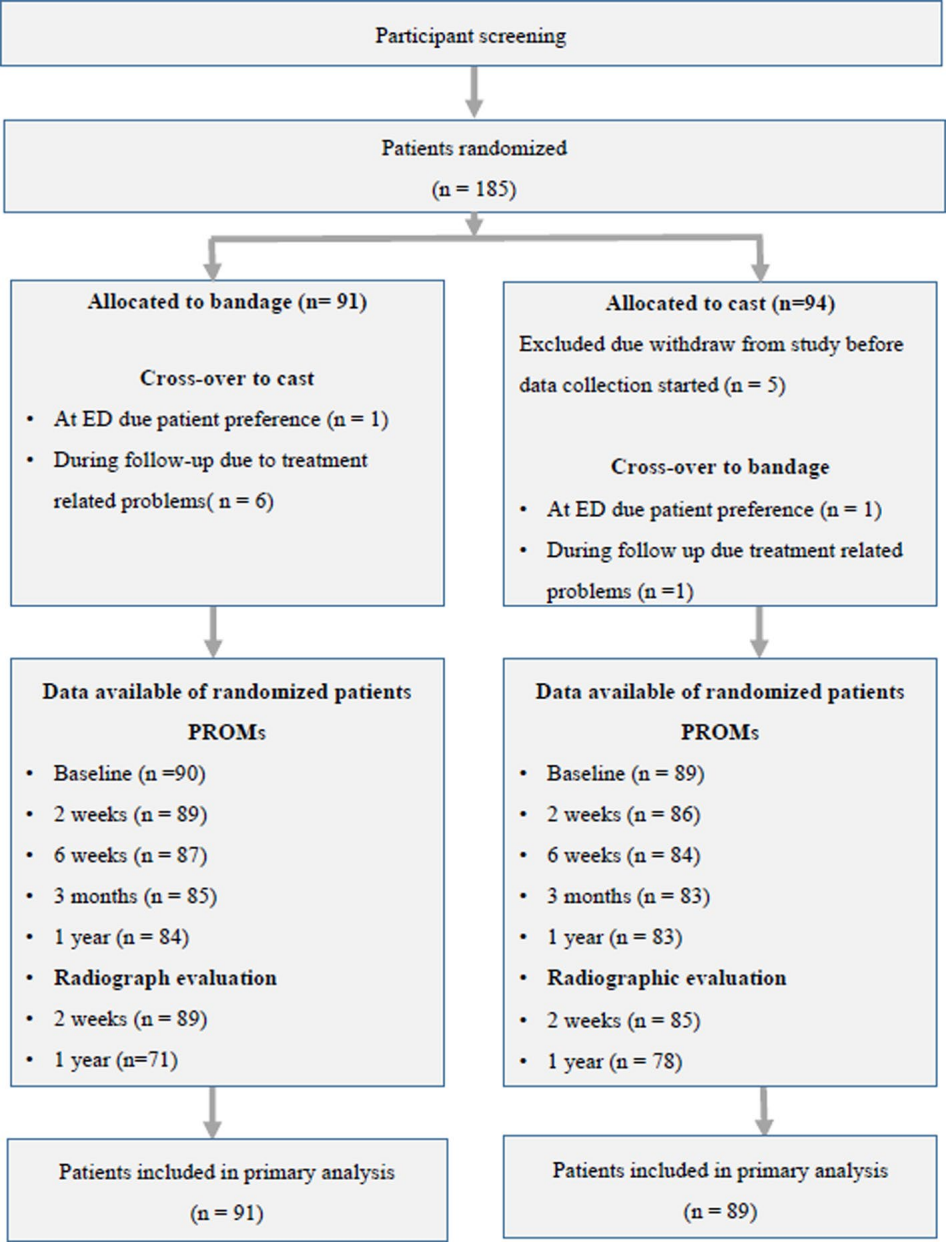
Study flow chart. Emergency department (ED). Patient-reported outcome measures (PROM)

Following inclusion, seven patients changed from bandaging to casting and two crossed-over from casting to bandaging within 6 days (Fig. 2). Baseline characteristics are reported in Table 2. Details about the treatment until the outpatient department appointment, after a mean of 13 days (SD ± 2 days), and follow-up thereafter, are reported in Table 3. Eventually, 16 patients (9%) were diagnosed with a scaphoid fracture. Fifteen were diagnosed with scaphoid fractures after 2 weeks and treated accordingly with a cast. One patient was primarily diagnosed with a wrist sprain, but owing to persistent pain after 3 months, computed tomography (CT) scanning demonstrated a scaphoid fracture. The patient was treated with a splint and union was established.

**Table 2:** Baseline characteristics of the 180 included patients

	Bandaging group (*n* = 91)	Casting group (*n* = 89)
Age (years), mean (SD)	45 (21)	45.3 (19)
Sex, *n* (%)		
Female	50 (55)	45 (51)
Male	41 (45)	44 (49)
Body mass index (kg/m^2^), mean (SD)	25.3 (4.6)	25.1 (4.1)
Upper extremity diseases or injuries, *n* (%)		
In medical history	40 (44)	51 (57)
Concomitant contralateral injury	4 (4)	2 (2)
Unknown	2 (2)	4 (4)
Diabetes mellitus, *n* (%)	4 (4)	4 (4)
Currently smoking, *n* (%)		
Yes	26 (29)	25 (28)
No	63 (69)	60 (68)
Unknown	2 (2)	4 (4)
Dominant side affected, *n* (%)		
Yes	46 (51)	43 (49)
No	43 (47)	42 (47)
Unknown	2 (2)	4 (4)
Trauma mechanism, *n* (%)		
Fall from height	7 (8)	5 (6)
Fall from standing	28 (31)	29 (33)
Road traffic accident	19 (21)	17 (19)
Sports injury	29 (32)	28 (31)
Fight or assault	4 (4)	7 (8)
Other	2 (2)	3 (3)
Unknown	2 (2)	0

**Table 3:** Secondary outcomes regarding follow-up, treatment, and diagnosis

	Bandage group (*n* = 91)	Cast group (*n* = 89)	*p*-Value
Number of additional hospital visits between ED and outpatient department appointment, *n* (%)			0.293
0	80 (88)	79 (89)	
1 or 2	11 (12)	10 (11)	
Treatment days between ED and outpatient department appointment, mean (sd)	5.3 (4.1)	12.1 (2.7)	** < 0,001**
Received treatment after outpatient department appointment, *n* (%)			0.92
Removable splint	6 (7)	6 (7)	
Cast	16 (18)	17 (19)	
No treatment	66 (73)	63 (71)	
Additional imaging, *n* (%)			
Radiographs	12 (13)	10 (11)	0.33
CT	10 (11)	14 (16)	0.47
MRI	0	2 (2)	0.47
Bonescan	0	1 (1)	0.99
Eventual diagnosis, *n* (%)			0.82
Wrist sprain	77 (85)	69 (81)	
Scaphoid fracture	7 (8)	9 (10)	
Other fracture	3 (3)	4 (4)	
(Suspected) soft tissue injury	2 (2)	3 (3)	
Unknown	2 (2)	4 (4)	
(Serious) adverse events during follow-up, *n* (%)			
Treatment-related problems	10 (11)	12 (13)	0.78
Persisting pain after 6 weeks	23 (26)	19 (24)	0.69
Persisting pain after 3 months	13 (16)	9 (11)	0.55
Persisting pain after 1 year	14 (11)	14 (18)	0.80

Bold value indicate *P*<0.001

ED, emergency department

### Primary outcome

The adjusted estimated mean QDASH score at 3 months was 8.7 (95% CI 6.3–12.1) in the bandaging group and 6.5 (95% CI 4.7–8.9) in the casting group (Table 4). At 3 months, 34 patients allocated to the bandaging group and 35 patients from the casting group reported the best possible QDASH score. Bandaging was noninferior to casting at 3 months, since the adjusted estimated difference between the two groups was 0.30 (95% CI −0.02–0.62) on the log-normal scale, which did not include the margin of noninferiority of 2.0 (Supplementary Table 1).

**Table 4:** Primary and secondary outcomes regarding as-randomized analysis of patient-reported functional outcome, pain, satisfaction with the treatment, wrist movement, and grip strength

	Adjusted estimated mean (95% CI)		Statistical outcome bandaging group compared with casting group
	Bandaging group	Casting group	
QDASH^$^			
After inclusion	59.6 (47.2 to 75.2)	62.7 (49.9 to 78.8)	^◊^
2 weeks	44.3 (35.1 to 56.0)	44.2 (35.4 to 55.3)	^◊^
6 weeks	24.6 (19.1 to 31.6)	22.0 (17.4 to 27.9)	^◊^
3 months	8.7 (6.3 to 12.1)	6.5 (4.7 to 8.9)	Noninferior
1 year	3.7 (2.4 to 5.5)	3.6 (2.5 to 5.3)	^◊^
PRWHE^$^			
After inclusion	78.2 (62.6 to 97.7)	82.1 (66.3 to 100^*^)	^◊^
2 weeks	59.3 (47.8 to 73.7)	59.9 (48.9 to 73.4)	^◊^
6 weeks	34.1 (27.2 to 42.8)	31.9 (26.0 to 39.1)	^◊^
3 months	13.0 (9.7 to 17.4)	10.6 (8.0 to 13.9)	^◊^
1 year	4.8 (3.3 to 7.0)	4.2 (3 to 5.9)	^◊^
VAS pain at rest^#^			
After inclusion	3.6 (2.7 to 4.9)	2.8 (2.1 to 3.8)	^◊^
2 weeks	2.6 (2.0 to 3.5)	2.1 (1.6 to 2.8)	^◊^
6 weeks	1.4 (1.0 to 1.9)	1.2 (0.9 to 1.7)	^◊^
3 months	0.4 (0.3 to 0.8)	0.4 (0.3 to 0.7)	^◊^
1 year	0.1 (0.1 to 0.3)	0.2 (0.1 to 0.4)	^◊^
VAS pain during movement^#^			
After inclusion	6.9 (5.7 to 8.3)	6.0 (5.0 to 7.2)	^◊^
2 weeks	5.6 (4.7 to 6.8)	4.9 (4.1 to 5.8)	^◊^
6 weeks	3.8 (3.1 to 4.6)	3.2 (2.6 to 3.9)	^◊^
3 months	1.9 (1.4 to 2.5)	1.6 (1.2 to 2.1)	^◊^
1 year	0.7 (0.5 to 1.0)	0.9 (0.6 to 1.4)	^◊^
Physical examination^∞^			
Palmar flexion			
2 weeks	8.6 (5.7 to 11.5)	14.5 (11.6 to 17.4)	*p* < 0.001
1 year	3.0 (−0.2 to 6.2)	1.4 (−1.7 to 4.4)	*p* = 0.38^◊^
Dorsal flexion			
2 weeks	5.7 (3.1 to 8.4)	12.3 (9.7 to 15.0)	*p* < 0.001
1 year	0.8 (−2.1 to 3.7)	0.6 (−2.2 to 3.3)	*p* = 0.88^◊^
Pronation			
2 weeks	0.5 (−0.7 to 1.8)	1.9 (0.7 to 3.2)	*p* = 0.06^◊^
1 year	−0.07 (−1.4 to 1.3)	0.2 (−1.1 to 1.5)	*p* = 0.74^◊^
Supination			
2 weeks	1.0 (−1.5 to 3.6)	4.3 (1.8 to 6.8)	*p* = 0.001
1 year	1.3 (−1.3 to 3.9)	1.0 (−1.6 to 3.6)	*p* = 0.79^◊^
Radial deviation			
2 weeks	3.1 (0.4 to 5.7)	3.8 (1.1 to 6.5)	*p* = 0.64^◊^
1 year	−2.0 (−5.0 to 1.0)	0.4 (−2.4 to 3.3)	*p* = 0.18^◊^
Ulnar deviation			
2 weeks	3.5 (2.2 to 4.8)	6.7 (5.4 to 8.0)	*p* < 0.001
1 year	1.2 (−0.2 to 2.6)	1.2 (−0.2 to 2.5)	*p* = 0.99^◊^
Grip strength			
2 weeks	11.9 (9.0 to 14.7)	11.1 (8.2 to 13.9)	*p* = 0.57^◊^
1 year	2.7 (−0.3 to 5.8)	1.7 (−1.3 to 4.7)	*p* = 0.50^◊^
Patient satisfaction^╪^			
2 weeks	7.3 (6.6 to 8.0)	6.4 (5.7 to 7.1)	*p* = 0.03
3 months	7.4 (6.7 to 8.1)	6.6 (5.9 to 7.3)	*p* = 0.049

QDASH, PRWHE, and VAS were analyzed with hurdle models specified as follows: randomized treatment, interaction term time by treatment, age, gender, and the presence of comorbidities that influence the function of the arms as fixed effects, and patients as a random effect. Hurdle models are two-part models, where the zero parts are modeled separately from the nonzero parts. In the hurdle models in the manuscript we assumed the nonzero part to follow a log-normal distribution. We created one model to analyze all outcomes up to 3 months with all measurements up to 3 months included (after inclusion, 2 weeks, 6 weeks, and 3 months) and one model to analyze 1-year outcomes with all outcomes available included (after inclusion, 2 weeks, 6 weeks, 3 months, and 1 year). Satisfaction and physical examination were analyzed with a linear mixed model specified as follows: randomized treatment, interaction term time by treatment, age, gender and the presence of comorbidities that influence the function of the arms as fixed effects, and patients nested with hospital as a random effect. We created one model including both time points. Statistical outcomes between the two groups are stated on the basis of the following criteria: (1) QDASH at 3 months if the 95% confidence interval (CI) of the adjusted estimated difference between the two groups included 2.0 (margin of noninferiority), and (2) QDASH (except at 3 months), PRWHE, and VAS if the 95% CI of the adjusted estimated difference included 0, and all other outcomes based on a *p*-value < 0.05. The adjusted estimated differences of all outcomes are presented in Supplementary Table 1

QDASH, Quick Disabilities of the Arm, Shoulder, and Hand; PRWHE, patient rated hand/wrist evaluation; VAS, visual analog scale

^*^Upper limit of the confidence interval was capped at the maximum score

^$^Scores range from 0 to 100 (0 best possible score).

^#^Score ranges from 0 to 10 (0 best possible score).

^╪^Score ranges from 0 to 10 (10 best possible score).

^∞^Physical examination is presented as degrees measuring the uninjured wrist compared with the injured wrist (lower score indicates a better physical functioning). Range of motion is expressed in degrees, grip strength in kilograms.

^◊^No statistically significant difference based on the adjusted estimated difference between the two groups on a log-normal scale calculated with a hurdle model

### Secondary outcomes

The QDASH score, PRWHE score, and VAS pain were not significantly different between the groups at any of the follow-up points (Table 4). Palmar flexion (*p* < 0.001), dorsal flexion (*p* < 0.001), supination (*p* = 0.001), and ulnar deviation (*p* < 0.001) were significantly better after 2 weeks in the bandaging group compared with the casting group. After 1 year, wrist range of motion did not differ significantly between the groups. Grip strength and finger motion did not differ between the bandaging and casting group after 2 weeks or 1 year (Supplementary Table 2).

Patients were significantly more satisfied with the allocated treatment in the bandaging group after 2 weeks (*p* = 0.03) and 3 months (*p* = 0.049) than the casting group. Most patients would have preferred bandaging at the emergency department (80% of bandaging group and 66% of casting group) when we evaluated this after 2 weeks, and 80% and 51% after 3 months.

### (Serious) adverse events

Treatment-related problems did not differ between the groups (*p* = 0.78, Table 3). Of the ten patients (11%) in the bandaging group who reported swollen and/or tingling fingers (*n* = 4) or too much pain (*n* = 6), the bandage was renewed in 4 patients, and 6 patients crossed over to casting. Twelve patients (13%) from the casting group reported pressure sores (*n* = 1); numb, tingling, or swollen fingers (*n* = 5); loosening of the cast (*n* = 3); wet cast (*n* = 1); or insufficient cast for holiday (*n* = 2). The cast was renewed in eight patients, and one patient changed to bandaging.

After 6 weeks, persistent pain was reported (at any time point) by 32 patients in the bandaging group and 31 in the casting group. The number of patients that reported persistent pain was not different between the groups at 6 weeks (*p* = 0.69), 3 months (*p* = 0.55), or 1 year (*p* = 0.80). The VAS pain scores of patients with persistent pain during follow-up from the 6-week time point was not different between the groups (Supplementary Table 3). None of the patients had scaphoid nonunion on the 1-year radiographs, and there were no other (serious) adverse events reported.

### As-treated comparison

As-treated analyses of all outcomes showed similar results as the as-randomized analyses, except for VAS pain after 1 year, which was better in the bandaging group compared with the casting group (Supplementary Table 4).

## Discussion

Only 10% of patients with suspected scaphoid fracture (but normal initial radiographs) actually have a fracture during follow-up, so 90% undergo unnecessary casting. Our pragmatic multicenter randomized controlled trial shows that bandaging resulted in noninferior functional outcomes at 3 months compared with casting. There was no difference between the groups regarding patient-reported functional outcomes, pain, or complications. The bandaging group had better wrist motion after 2 weeks and were more satisfied with the treatment. Bandaging seems safe as there were no scaphoid nonunions or other serious adverse events.

Our results regarding adverse events and pain are similar to those of Sjølin and Andersen, who also compared bandaging with casting in patients with suspected scaphoid fracture and normal initial radiographs [7]. In line with our findings, the treatment of sprains, and even some fractures, is already shifting from casting to bandaging with early mobilization to prevent stiffness [2, 18, 19].

In total, 9% of our patients were diagnosed with a scaphoid fracture during follow-up and treated conservatively accordingly. The current overtreatment of patients with suspected fracture attempts to avoid nonunion and the subsequent risk of progressive osteoarthritis; however, the absence of nonunions after 1 year shows that bandaging seems safe, with the reexamination approach after 2 weeks. This is supported by Langhoff et al. [6] who reported similar union rates when adequate treatment was started within 4 weeks. Occult scaphoid fractures are mainly stable waist or distal pole fractures, which are less likely to result in nonunion [20–22]. Studies reporting outcome of adequately treated occult scaphoid fractures with cast or surgery according to the guidelines for scaphoid fractures do not report any scaphoid nonunions [7, 23, 24].

While delayed reexamination is a commonly used approach, an alternative is to perform advanced imaging during the initial consultation to give an immediate diagnosis [25]. Recently, studies have focused on additional imaging, but there is no reference standard for diagnosing occult scaphoid fractures,because each modality has different drawbacks [4, 26–28]. CT is less accurate, with a risk of false negative results [negative predictive value (94 ± SD 5)] [29]. Magnetic resonance imaging (MRI) has the highest accuracy, but can give false positive results [positive predictive value (95 ± SD 12)]. In the Netherlands, the standard care for suspected scaphoid fractures is cast and a reexamination after 2 weeks with a radiograph in a symptomatic patient. Some guidelines such as the National Institute for Health and Care Excellence (NICE) guidance from the UK, advises an MRI direct from the emergency department; however, only 13% of the hospitals have an MRI directly available [30]. Advanced imaging is not readily available in many countries making implementation challenging [31, 32]. Bandages are readily available in most global healthcare settings.

Our study has some limitations. Firstly, there is no prespecified reference standard for defining the presence of a scaphoid fracture. Imaging modalities such as radiographs, MRI, and CT scans are used to identify scaphoid fractures all with their own downsides. Within our study, an occult scaphoid fracture was defined according to the treating physician on the basis of their interpretation of the physical examination, radiographs, and/or further imaging. Only radiographs at the outpatient clinic were necessary for this study. This pragmatic approach makes our results transferable to clinical practice.

Secondly, 1-year radiographs were only obtained in 83% of the patients owing to the coronavirus disease (COVID-19) pandemic, but 93% completed their 1-year questionnaires. Previous research showed that patient-reported outcomes in those with hand and wrist complaints collected during the pandemic can be used in clinical research and were not affected by restrictions or altered behavior owing to lockdown [33].

Thirdly, our study was not powered to detect a difference in nonunion rates between the treatment approaches. Given the extremely low nonunion rate, future randomized controlled trials to examine this seem impossible [7, 23, 24]. We chose a 1-year follow-up to identify scaphoid nonunions on radiographs. Most studies define a scaphoid nonunion as failed union, minimally 3–6 months after the trauma on the basis of radiographs, MRI, or CT [34–36]. Previously, it has been suggested that radiographs at least 6–12 months after the injury are necessary to identify scaphoid nonunions [37, 38]. Perhaps a longer follow-up or another imaging modality is preferred, such as CT; however, to our knowledge, little is known in literature, and most studies do not use a longer follow-up than 1 year to address union for scaphoid fractures. A longer follow-up could be used to review patient-reported outcomes over a longer period of time or to identify arthritic changes in the wrist.

Fourthly, we used patient-reported outcomes as our primary outcome and as some secondary outcomes within our study. Patient-reported outcomes could not be blinded in our study, since patients are aware if they received below-elbow bandage or a below-elbow cast. However, physical examination was performed unblinded owing to practical issues.

In summary, our pragmatic randomized controlled trial found that functional outcomes at 3 months using bandaging were noninferior compared with casting. There was also no difference in patient-reported functional outcomes, pain, or complications between the two groups during follow-up. Patients in the bandaging group had better wrist range of motion after 2 weeks and were more satisfied. We suggest that bandaging seems to be an alternative to casting in suspected scaphoid fractures, when initial radiographs are normal, and reexamination is performed after 2 weeks.

## Supplementary Information

Below is the link to the electronic supplementary material.

Supplementary file1 (DOCX 35 KB)

## Data Availability

Individual de-identified participant data that underlie the results reported in this paper (text, tables, figures, and appendices) and the study protocol will be shared upon request. Data will be available, beginning from 12 months and ending after 5 years, following publication of this paper. Data will be available for researchers who provide a methodologically sound scientific proposal, which has been approved by an ethical committee. Proof of the latter should be provided. Analyses should achieve the aims as reported in the approved proposal. Proposals for data should be directed to m.reijman@erasmusmc.nl.

## Abbreviations

ED: Emergency department
QDASH: Quick Disabilities of the Arm, Shoulder, and Hand
PRWHE: Patient-Rated Hand/Wrist Evaluation Score
VAS: Visual analog scale
SUSPECT study: Clinically SUSPEcted SCaphoid fracTure: treatment with supportive bandage or cast study
Castor EDC: Castor electronic data capture
