# Supplementary material for: Can we avoid casting for suspected scaphoid fractures? A multicenter randomized controlled trial
Source: J Orthop Traumatol. 2025 Mar 5;26:14. doi: 10.1186/s10195-025-00822-5 (PMC11883040; doi:10.1186/s10195-025-00822-5)
Supplement: Supplementary file 1 — Supplementary file1 (DOCX 35 KB) [file 10195_2025_822_MOESM1_ESM.docx]

**Supplementary table 1.** Adjusted estimated difference between the two groups with the as-randomized analyses of the patient-reported functional outcome, pain, satisfaction with the treatment, wrist motion, and grip strength

|  | Adjusted estimated difference  (bandage compared to cast) |  |  |  |
| --- | --- | --- | --- | --- |
| QDASH^$^ | | |  | |
| After Inclusion | | -0.05 (-0.28 to 0.18) |  |  |
| 2 weeks | | 0.002 (-0.23 to 0.23) |  |  |
| 6 weeks | | 0.11 (-0.13 to 0.35) |  |  |
| 3 months | | 0.30 (-0.02 to 0.62) |  |  |
| 1 year | | 0.004 (-0.39 to 0.39) |  |  |
| PRWHE^$^ | | |  | |
| After inclusion | | -0.05 (-0.27 to 0.17) |  |  |
| 2 weeks | | -0.009 (-0.22 to 0.20) |  |  |
| 6 weeks | | 0.07 (-0.15 to 0.28) |  |  |
| 3 months | | 0.21 (-0.08 to 0.49) |  |  |
| 1 year | | 0.13 (-0.22 to 0.49) |  |  |
| VAS pain at rest^#^ | | |  |  |
| After inclusion | | 0.25 (-0.05 to 0.56) |  |  |
| 2 weeks | | 0.21 (-0.08 to 0.51) |  |  |
| 6 weeks | | 0.13 (-0.20 to 0.46) |  |  |
| 3 months | | -0.01 (-0.52 to 0.49) |  |  |
| 1 year | | -0.38 (-1.17 to 0.42) |  |  |
| VAS pain during movement^#^ | | |  |  |
| After inclusion | | 0.14 (-0.05 to 0.32) |  |  |
| 2 weeks | | 0.14 (-0.04 to 0.33) |  |  |
| 6 weeks | | 0.15 (-0.05 to 0.36) |  |  |
| 3 months | | 0.17 (-0.23 to 0.47) |  |  |
| 1 year | | -0.28 (-0.71 to 0.14) |  |  |
| Physical examination^∞^ | | |  |  |
| Palmar flexion | | |  |  |
| 2 weeks | | -5.9 (-9.3 to -2.5) |  |  |
| 1 year | | 1.6 (-2.0 to 5.3) |  |  |
| Dorsal flexion | | |  |  |
| 2 weeks | | -6.6 (-9.7 to -3.5) |  |  |
| 1 year | | 0.3 (-3.1 to 3.6) |  |  |
| Pronation | | |  |  |
| 2 weeks | | -1.4 (-2.9 to 0.1) |  |  |
| 1 year | | -0.3 (-1.9 to 1.4) |  |  |
| Supination | | |  |  |
| 2 weeks | | -3.3 (-5.2 to -1.3) |  |  |
| 1 year | | 0.3 (-1.8 to 2.4) |  |  |
|  | | |  |  |
| 2 weeks | | -0.8 (-4.1 to 2.5) |  |  |
| 1 year | | -2.4 (-6.0 to 1.1) |  |  |
| Ulnar deviation | | |  |  |
| 2 weeks | | -3.2 (-4.8 to -1.7) |  |  |
| 1 year | | 0.0 (-1.7 to 1.7) |  |  |
| Grip strength | | |  |  |
| 2 weeks | | 0.8 (-2.0 to 3.6) |  |  |
| 1 year | | 1.0 (-2.0 to 4.0) |  |  |
| Patient satisfaction^ⱡ^ | | |  |  |
| 2 weeks | | 0.9 (0.1 to 1.7) |  |  |
| 3 months | | 0.8 (0.004 to 1.6) |  |  |

QDASH = Quick Disabilities of the arm, shoulder and hand. PRWHE = Patient Rated Hand/Wrist Evaluation. VAS = Visual Analog Scale.

QDASH, PRWHE, VAS are analyzed with hurdle models specified as follows: randomized treatment, interaction term time by treatment, age, gender and the presence of comorbidities that influence the function of the arms as fixed effects, patients as a random effect. Hurdle models are two-part models, where the zero are modelled separately from the non-zero part. In the hurdle models in the manuscript we assumed the non-zero part to follow a lognormal distribution. We created one model created to analyze all outcomes up to three months with all measurements up to 3 months included (after inclusion, 2 weeks, 6 weeks, 3 months) and one model to analyze 1 year outcome with all outcomes available included (after inclusion, 2 weeks, 6 weeks, 3 months and 1 year). Estimated size of difference is expressed on a lognormal scale for these outcomes.

Satisfaction and physical examination are analyzed with a linear mixed model specified as follows: randomized treatment, interaction term time by treatment, age, gender and the presence of comorbidities that influence the function of the arms as fixed effects, patients nested with hospital as a random intercept. We created one model including both time points.

^$^ Scores range from 0 to 100 (0 best possible score)

^#^ Score ranges from 0 to 10 (0 best possible score)

^ⱡ^ Score ranges from 0 to 10 (10 best possible score)

^∞^ Physical examination is presented as measuring the uninjured wrist compared to the injured wrist (lower score indicates a better score). Range of motion is expressed in degrees, grip strength in kilograms.

**Supplementary tale 2**. As-randomized analyses and as-treated analyses of finger motion

|  | As-randomized analyses | | | | As-treated analyses | | |  |
| --- | --- | --- | --- | --- | --- | --- | --- | --- |
|  | **Adjusted estimated mean (95% CI)** | | **Adjusted estimated difference**  **(95% CI)** | **p- value** | **Adjusted estimated mean (95% CI)** | | **Adjusted estimated difference**  **(95% CI**) |  |
|  | Bandaging group | Casting group |  |  | Bandaging group | Casting group |  |  |
| Kapandji score^#^ | | | | | | | | |
| 2 weeks | | 0.7 (0.4 to 1.0) | 1.0 (0.7 to 1.3) | -0.3 (-0.6 to 0.1) | 0.13 | 0.6 (0.3 to 0.9) | 1.1 (0.8 to 1.4) | -0.5 (-0.8 to -0.1) |
| 1 year | | 0.2 (-0.1 to 0.5) | 0.1 (-0.3 to 0.4) | 0.1 (-0.2 to 0.5) | 0.45 | 0.1 (-0.2 to 0.5) | 0.1 (-0.2 to 0.4) | 0.0 (-0.4 to 0.4) |
| Finger-palm distance dig II | | | | | | | | |
| 2 weeks | | -1.1 (-2.4 to 0.2) | -1.3 (-2.6 to 0.0) | 0.2 (-1.4 to 1.8) | 0.79 | -1.0 (-2.4 to 0.3) | -1.3 (-2.6 to 0.0) | 0.2 (-1.4 to 1.8) |
| 1 year | | -0.1 (-1.5 to 1.3) | -0.2 (-1.6 to 1.1) | 0.1 (-1.6 to 1.8) | 0.89 | -0.2 (-1.6 to 1.3) | -0.2 (-1.5 to 1.2) | 0.0 (-1.7 to 1.7) |
| Finger-palm distance dig III | | | | | | | | |
| 2 weeks | | -0.9 (-2.0 to 0.2) | -1.3 (-2.5 to -0.2) | 0.4 (-0.9 to 1.8) | 0.55 | -0.9 (-2.0 to 0.2) | -1.3 (-2.4 to -0.3) | 0.4 (-0.9 to 1.8) |
| 1 year | | -0.2 (-1.4 to 1.1) | -0.1 (-1.3 to 1.1) | 0.0 (-1.5 to 1.4) | 0.96 | -0.2 (-1.4 to 1.0) | -0.1 (-1.2 to 1.1) | -0.1 (-1.6 to 1.3) |
| Finger-palm distance dig IV | | | | | | | | |
| 2 weeks | | -1.0 (-2.2 to 0.1) | -1.1 (-2.3 to 0.1) | 0.1 (-1.4 to 1.5) | 0.93 | -1.0 (-2.2 to 0.2) | -1.1 (-2.2 to 0.1) | 0.1 (-1.4 to 1.5) |
| 1 year | | -0.2 (-1.5 to 1.1) | 0.2 (-1.0 to 1.5) | -0.4 (-1.9 to 1.1) | 0.61 | -0.3 (-1.6 to 1.1) | 0.3 (-0.9 to 1.5) | -0.5 (-2.1 to 1.0) |
| Finger-palm distance dig V | | | | | | | | |
| 2 weeks | | -0.9 (-2.0 to 0.1) | -1.4 (-2.4 to -0.3) | 0.4 (-0.8 to 1.7) | 0.49 | -0.9 (-2.0 to 0.2) | -1.4 (-2.4 to -0.4) | 0.5 (-0.8 to 1.7) |
| 1 year | | -0.2 (-1.4 to 0.9) | 0.1 (-1.0 to 1.2) | -0.4 (-1.7 to 1.0) | 0.58 | -0.3 (-1.4 to 0.9) | 1.2 (-0.9 to 1.3) | -0.5 (-1.8 to 0.9) |

The outcomes are analyzed with a linear mixed model specified as follows: randomized treatment (as-randomized analyses) or received treatment (as-treated analyses), interaction term time by treatment, age, gender and the presence of comorbidities that influence the function of the arms as fixed effects, patients nested with hospital as a random intercept. We created one model including both time points. P<0.05 is considered significant. Outcomes are presented as measuring the uninjured wrist compared to the injured wrist (lower score indicates a better score). Finger-palm distance is in millimeters.

^#^ Score ranges from 0 to 10 (10 best possible score)

**Supplementary table 3.** VAS pain during rest and activity of patients reporting persisting pain after 6 weeks (n=42), 3 months (n=22) or 1 year (n=28).

|  | | Bandaging group | Casting group | p-value |
| --- | --- | --- | --- | --- |
| Persisting pain after six weeks | **VAS pain rest, median [IQR]** | 1.0 [0.0 to 4.5] | 1.0 [0.0 to 2.5] | 0.63 |
|  | **VAS pain movement, median [IQR]** | 3.0 [2.0 to 5.0] | 4.5 [2.0 to 7.8] | 0.47 |
| Persisting pain after three months | **VAS pain rest, median [IQR]** | 4.0 [1.0 to 5.0] | 2.0 [0.0 to 3.0] | 0.21 |
|  | **VAS pain movement, median [IQR]** | 6.0 [6.0 to 8.0] | 7.0 [6.0 to 8.0] | 0.61 |
| Persisting pain after one year | **VAS pain rest, median [IQR]** | 1.0 [1.0 to 2.0] | 1.0 [1.0 to 2.0] | 0.67 |
|  | **VAS pain movement, median [IQR]** | 5.0 [3.5 to 7.0] | 4.0 [2.0 to 6.0] | 0.45 |

VAS = Visual Analog Scale

**Supplementary table 4.** As-treated analyses of the secondary outcomes patient-reported functional outcome, pain, patient satisfaction, wrist movement, and grip strength

|  | Adjusted estimated mean (95% CI) | | Adjusted estimated difference bandage group compared to cast group |  |
| --- | --- | --- | --- | --- |
|  | **Bandaging group** | **Casting group** |  |  |
| QDASH | | | |  |
| After Inclusion | 57.4 (45.7 to 72.2) | 65 (53.8 to 78.6) | -0.12 (-0.33 to 0.09) |  |
| 2 weeks | 42.7 (33.9 to 53.8) | 46.0 (38.3 to 55.2) | -0.07 (-0.28 to 0.13) |  |
| 6 weeks | 23.6 (18.3 to 30.4) | 23.0 (19.1 to 27.8) | 0.03 (-0.20 to 0.25) |  |
| 3 months | 8.4 (5.9 to11.8) | 6.9 (5.3 to 8.9) | 0.20 (-0.10 to 0.50) |  |
| 1 year | 3.5 (2.4 to 5.3) | 3.7 (2.7 to 5.2) | -0.06 (-0.43 to 0.31) |  |
| PRWHE | | | |  |
| After inclusion | 75.6 (59.5 to 96.2) | 85.0 (70.5 to 100^*^) | -0.12 (-0.33 to 0.10) |  |
| 2 weeks | 57.0 (45.3 to 71.7) | 62.4 (52.4 to 74.3) | -0.09 (-0.29 to 0.11) |  |
| 6 weeks | 32.3 (25.7 to 40.7) | 33.6 (28.2 to 40.0) | -0.04 (-0.23 to 0.17) |  |
| 3 months | 12.0 (8.9 to 16.1) | 11.4 (8.9 to 14.5) | 0.05 (-0.22 to 0.32) |  |
| 1 year | 4.3 (2.8 to 6.7) | 4.7 (3.2 to 6.7) | -0.08 (-0.48 to 0.32) |  |
| VAS pain at rest | | | |  |
| After inclusion | 3.5 (2.6 to 4.8) | 3.0 (2.2 to 4.0) | 0.18 (-0.12 to 0.48) |  |
| 2 weeks | 2.5 (1.9 to 3.4) | 2.2 (1.6 to 3.0) | 0.12 (-0.18 to 0.42) |  |
| 6 weeks | 1.3 (0.9 to 1.8) | 1.3 (0.9 to 1.8) | 0.004 (-0.34 to 0.35) |  |
| 3 months | 0.4 (0.2 to 0.7) | 0.5 (0.3 to 0.8) | -0.20 (-0.72 to 0.32) |  |
| 1 year | 0.1 (0.0 to 0.2) | 0.2 (0.1 to 0.5) | -1.24 (-2.22 to -0.26) |  |
| VAS pain during movement | | | |  |
| After inclusion | 6.6 (5.4 to 8.1) | 6.3 (5.2 to 7.6) | 0.06 (-0.14 to 0.26) |  |
| 2 weeks | 5.4 (4.4 to 6.6) | 5.1 (4.2 to 6.1) | 0.05 (-0.14 to 0.25) |  |
| 6 weeks | 3.6 (2.9 to 4.5) | 3.4 (2.8 to 4.1) | 0.05 (-0.16 to 0.26) |  |
| 3 months | 1.7 (1.3 to 2.4) | 1.7 (1.3 to 2.2) | 0.04 (-0.27 to 0.34) |  |
| 1 year | 0.6 (0.4 to 0.9) | 1.0 (0.7 to 1.5) | -0.55 (-0.97 to -0.14) |  |
| Physical examination | | | |  |
| Palmair flexion | | | |  |
| 2 weeks | 7.3 (4.4 to 10.2) | 15.3 (12.5 to 18.1) | -8.1 (-11.4 to -4.7) |  |
| 1 year | 3.6 (0.4 to 6.7) | 1.0 (-2.0 to 3.9) | 2.6 (-1.0 to 6.2) |  |
| Dorsal flexion | | | |  |
| 2 weeks | 5.2 (2.5 to 7.9) | 12.5 (9.9 to 15.1) | -7.4 (-10.4 to -4.3) |  |
| 1 year | 0.6 (-2.4 to 3.5) | 0.8 (-2.0 to 3.5) | -0.2 (-3.5 to 3.2) |  |
| Pronation | | | |  |
| 2 weeks | 0.6 (-0.6 to 1.9) | 1.8 (0.6 to 3.0) | -1.2 (-2.7 to 0.3) |  |
| 1 year | -0.01 (-1.4 to 1.4) | 0.1 (-1.1 to 1.4) | -0.2 (-1.8 to 1.5) |  |
| Supination | | | |  |
| 2 weeks | 1.0 (-1.6 to 3.6) | 4.1 (1.6 to 6.6) | -3.1 (-5.1 to -1.2) |  |
| 1 year | 1.2 (-1.5 to 3.9) | 1.1 (-1.5 to 3.6) | 0.1 (-2.0 to 2.3) |  |
| Radial deviation | | | |  |
| 2 weeks | 2.8 (0.1 to 5.6) | 4.0 (1.4 to 6.6) | -1.2 (-4.5 to 2.1) |  |
| 1 year | -2.0 (-5.0 to 1.1) | 0.4 (-2.4 to 3.2) | -2.3 (-5.9 to 1.2) |  |
| Ulnar deviation | | | |  |
| 2 weeks | 3.1 (1.8 to 4.4) | 6.9 (5.7 to 8.2) | -3.8 (-5.4 to -2.3) |  |
| 1 year | 1.0 (-0.5 to 2.4) | 1.3 (0.0 to 2.7) | -0.4 (-2.0 to 1.3) |  |
| Grip strength | | | |  |
| 2 weeks | 11.1 (8.2 to 14.0) | 11.8 (9.1 to 14.6) | -0.8 (-3.6 to 2.0) |  |
| 1 year | 2.1 (-0.9 to 5.2) | 2.2 (-0.7 to 5.1) | -0.1(-3.1 to 2.9) |  |
| Satisfaction with treatment | | | |  |
| 2 weeks | 7.6 (6.9 to 8.2) | 6.4 (5.8 to 7.1) | 1.2 (0.4 to 1.9) |  |
| 3 months | 7.7 (7.1 to 8.4) | 6.7 (6.0 to 7.3) | 1.1 (0.3 to 1.8) |  |

QDASH = Quick Disabilities of the arm, shoulder and hand. PRWHE = Patient Rated Hand/Wrist Evaluation. VAS = Visual Analog Scale

QDASH, PRWHE, VAS are analyzed with hurdle models specified as follows: received treatment, interaction term time by treatment, age, gender and the presence of comorbidities that influence the function of the arms as fixed effects, patients as a random effect. We created one model to analyze all outcomes up to 3 months with all measurements up to 3 months included (after inclusion, 2 weeks, 6 weeks, 3 months) and one model to analyze one year outcome with all outcomes available included (after inclusion, 2 weeks, 6 weeks, 3 months and 1 year).

Satisfaction and physical examination are analyzed with a linear mixed model specified as follows: randomized treatment, interaction term time by treatment, age, gender and the presence of comorbidities that influence the function of the arms as fixed effects, patients nested with hospital as a random intercept. We created one model including both time points.

Adjusted estimated differences of the QDASH, PRWHE, and VAS are the adjusted estimated differences based on a lognormal distribution.

^*^ Upper limit of the confidence interval was capt at the maximum score.

^$^ Scores range from 0 to 100 (0 best possible score)

^#^ Score ranges from 0 to 10 (0 best possible score)

^ⱡ^ Score ranges from 0 to 10 (10 best possible score)

^∞^ Physical examination is presented as measuring the uninjured wrist compared to the injured wrist (lower score indicates a better score). Range of motion is expressed in degrees, grip strength in kilograms.
